# Knowledge of occupational exposure to HIV: a cross sectional study of healthcare workers in Tumbi and Dodoma hospitals, Tanzania

**DOI:** 10.1186/s12913-015-0700-z

**Published:** 2015-01-22

**Authors:** Kijakazi Obed Mashoto, Godfrey Martin Mubyazi, Adiel K Mushi

**Affiliations:** National Institute for Medical Research, P.O. Box 9653, Dar es Salaam, Tanzania; Tanzania National Health Research Forum, P.O. Box 9653, Dar es Salaam, Tanzania

**Keywords:** Knowledge, Occupational exposure, HIV transmission, Healthcare workers

## Abstract

**Background:**

Insufficient knowledge on blood-borne pathogens has been identified as a factor that influences occupational exposure to needle stick and sharps injuries. The objective of this study was to assess healthcare workers’ knowledge on occupational exposure to HIV.

**Methods:**

A cross sectional survey was conducted at Tumbi designated regional hospital and Dodoma regional hospital, Tanzania in February 2012. A self-administered questionnaire was used to capture information on knowledge of occupational exposure to HIV infection.

**Results:**

A total of 401 healthcare workers responded to a self-administered questionnaire. High proportion of healthcare workers (96.3%) understood that they are at risk of occupational exposure to HIV. The majority of healthcare workers trained on post exposure prophylaxis procedure and use of personal protective equipment were clinicians (87.1% and 71.4% respectively) and nurses (81.8% and 74.6% respectively). Over a quarter of the healthcare workers were not aware of whom to contact in the event of occupational exposure. One third of healthcare workers did not have comprehensive knowledge on causes of occupational HIV transmission and did not know when post exposure prophylaxis is indicated. Healthcare workers not trained on the use of person protective equipment were less likely to have comprehensive knowledge on occupational exposure to HIV (OR = 0.5; 95% CI 0.3 – 0.9). Knowledge on causes of occupational exposure varied with the cadre of healthcare workers. Nurses were more likely to have comprehensive knowledge on occupational exposure to HIV than non-clinical staff (OR = 2.6; 95% CI 1.5 – 4.5).

**Conclusion:**

A substantial proportion of studied healthcare workers had little knowledge on occupational exposure to HIV and was not aware of a contact person in the event of occupational exposure to HIV. Training on post exposure prophylaxis and infection prevention and control including the use of person protective equipment provided to nurses and clinicians should be extended to other clinical and non-clinical hospital staff.

## Background

Health care workers (HCWs) are potentially exposed to occupational HIV infections through injuries/accidents from sharp objects such as needle stick, scissors and knives or contact with blood or other infectious body fluids. Potential risk of occupational HIV transmission increases with unsafe practices such as careless handling of contaminated needles, unnecessary injections on demand, reuse of inadequately sterilized needles, improper handling and disposal of hazardous waste [1,2]. Other factors that contribute to the risk of occupational HIV transmission in developing countries include lack of stringent safety procedures and standards at work place, limited resources for post exposure evaluation and treatment, high rates of undiagnosed HIV infection and limited access to personal protective equipment [3-5]. All these can increase the risk of occupational exposure to blood borne pathogens including HIV.

In order to prevent transmission of pathogens after potential exposure and also to refer for comprehensive management to minimize the risk of infection after potential exposure to HIV, post exposure prophylaxis (PEP) is needed [6]. PEP includes first aid, counseling, risk assessment, laboratory investigations based on the informed consent of the exposed person and source. Following risk assessment, a short term course of antiretroviral drugs is given for 28 days, along with a follow-up evaluation [7].

Evidence shows that hospitals can overcome challenge in protecting both the HCWs and patient population by improving HCWs knowledge of blood borne pathogen transmission and post-exposure management through educational initiatives. A study conducted in Dar es Salaam, Tanzania revealed that approximately 69% of exposed HCWs immediately cleaned their wounds and sought professional help. This suggests that the majority of the HCWs were aware of the risk of HIV transmission at workplace [8]. Studies have indicated that the risk of occupational HIV transmission is well recognized by HCWs [9-11]. However, another study reported that HCWs have inadequate knowledge of PEP for occupational exposure to HIV [12].

A study conducted in Tumbi and Dodoma hospitals, Tanzania reported the prevalence of occupational exposure to HIV among HCWs stand at 47.9% [13]. The prevalence of HIV among trauma patients alone in Bugando hospital Tanzania is 11.6% which places HCWs at a substantial risk of exposure to HIV [14]. However, in Tanzania the effort to characterize the risk of HIV infection acquisition by HCWs is hampered by poor quality of hospital records [15].

Insufficient knowledge of risk of contamination and preventive measures for blood-borne pathogens influences occupational exposure to needle stick and sharps injuries [16]. Consequently national guidelines for infection prevention and control were developed with the objective to protect HCWs and patients from occupational infection [7]. Nevertheless, research evidence on the knowledge of occupational HIV transmission among HCWs is still limited in Tanzania. This paper presents results from a survey which was conducted in two regional hospitals to assess HCWs knowledge on occupational exposure to HIV.

## Methods

### Study design and area

This study was conducted in two regions located long distances from each other in Tanzania. One regional hospital was selected in each region namely, Tumbi Regional Hospital in Coast region near Dar es Salaam the commercial capital of Tanzania and Dodoma Regional Hospital in Dodoma Region. Besides the routine and referral services given to patients from within and beyond their catchment areas, Tumbi hospital has potentially higher chances of exposure to occupational hazards due to the fact that it is located near a road in an area with relatively higher numbers of road accidents, it serves people from different regions in Tanzania who encounter accidents on their way to or from Dar es Salaam. Dodoma hospital was selected to represent hospitals which receive low numbers of road accident cases [13]. Both hospitals have a focal person to contact in case of occupational exposure to HIV.

### Study population and sample size

Study participants included medical and dental specialists, dental officers, assistant medical and dental officers, clinical officers, assistant clinical officers, laboratory technicians and technologists, radiologists, radiographers, physiotherapists, nurses and health attendants/hospital cleaners. The sample size was determined using the following formula n = Z^2^ p (1-p)/d^2^ where, **Z =** value at a specified confidence level, **P =** approximate proportion of the event in the population and **d =** acceptable margin of error in estimating the true population proportion [17]. The proportion of Tanzanian HCWs with adequate knowledge on occupational exposure to HIV is 35% [18] and given 1.96 value of the 95% confidence interval and 0.04 an acceptable margin of error, and taking into consideration of 10% non-response, the minimum sample size required for the study was 600. Due to low number of HCWs employed at the selected hospital, all 751 HCWs in the two hospitals were invited to participate in the study (Dodoma = 391 and Tumbi = 360).

### Data collection

A self-administered questionnaire was designed to capture HCW’s knowledge on HIV transmission, general knowledge on occupational exposure to HIV, causes and prevention of occupational exposure to HIV (see attached questionnaire). Data collection methods are fully described elsewhere [13].

Respondents were asked if they knew when use of post exposure prophylaxis (PEP) is recommended and whom to contact in the event of occupational exposure to HIV. Knowledge on prevention of occupational exposure to HIV was assessed by an open ended question. Age, sex, professional training attained and type of cadre were the socio-demographic variables included in the questionnaire. During data collection, four research assistants were stationed at each hospital to ensure close follow up of the data including giving reminders when deemed necessary. The respondents were asked to return the duly filled-in questionnaires at their convenience within the agreed time to the researchers. The returned questionnaires were checked to assess accuracy and completeness. In case of any missing variables/data or errors the questionnaire was returned back to the respondent to make indicated corrections.

### Data analysis and statistical techniques

Data were entered into Epi-info and analysis was performed using SPSS version 17. Bivariate associations were tested using Chi-square. Association of knowledge on occupational exposure to HIV with socio-demographic factors and education on infection prevention and control was tested by multinomial logistic regression.

Knowledge on HIV transmission was assessed by asking HCWs to identify body fluids that are considered to be of high and low risk for HIV transmission. The correct answer was given score 1 and incorrect answer was given score 0. Total score on knowledge of HIV transmission was constructed by summation of 7 items and then dichotomized into little (0) and comprehensive (1) knowledge.

General knowledge on occupational exposure to HIV was assessed by 14 statements scored using a 4-point Likert scale. The scores were then dichotomized into 1 = agreed and 0 = disagreed. Total score of the general knowledge on occupational exposure was obtained by summation of the 14 items and the dichotomization into little (0) and comprehensive (1) knowledge.

Knowledge on causes of occupational exposure to HIV was assessed by asking HCWs to identify causes from a list of 6 possible causes. Each correct identification scored 1 and incorrect identification scored 0. Total knowledge score on causes of occupational exposure was constructed by summation of the 6 items and the dichotomized into little (0) and comprehensive (1) knowledge.

Knowledge on when the use of PEP is indicated was assessed by posing four scenarios for which HCW was required to indicate whether PEP is recommended or not for each scenario. The correct response was given score 1 and the incorrect response was given score 0. Total knowledge score on PEP indication was obtained by summing the responses of the four scenarios. The score was then dichotomized into little (0) and comprehensive (1) knowledge.

Knowledge on whom to contact in event of occupational exposure to HIV was assessed by a single question. HCWs were asked if they knew of a person to contact in the event of occupational exposure to HIV. Response options were 1 = Yes and 0 = No.

Final knowledge score on occupational exposure to HIV was computed as the sum of knowledge on HIV transmission, general knowledge on occupational exposure to HIV infections, knowledge on causes of occupational exposure to HIV, knowledge on when PEP is indicated, knowing whom to contact in the event of occupational exposure and perceived risk of occupational HIV transmission. The score was then dichotomised into 0 = little knowledge and 1 = comprehensive knowledge

For analysis purposes, type of HCWs cadre was categorised into four groups. The first group was referred to as clinicians to include medical/dental specialists, medical and dental officers, assistant medical and dental officers, medical/dental assistants and clinical officers. The second group was referred to as nurses to include all registered, enrolled, enlisted and assistant nurses. The third group was other clinical staff, which comprised of pharmacists, assistant pharmacists, pharmaceutical technicians, laboratory technicians, laboratory technologists, health attendants/hospital cleaners, physiotherapists and radiologists. Health attendants also serve as hospital cleaners and therefore were grouped with other clinical staff. The fourth group is non-clinical staff and consisted of record keepers, social welfare officers, health officers, health secretaries and others. Professional training attained was re-categorised into 1 = Degree, 2 = Diploma (including the original option 2 = advance diploma and 3 = diploma) and 3 = Certificate (combining original option 4 and 5 for certificate and other respectively).

### Ethical considerations

Ethical clearance was obtained from the Medical Research Coordinating Committee (MRCC) through the National Institute for Medical Research (NIMR) Secretariat. Regional and District/Municipal Government and Health Authorities for Coast and Dodoma Regions also gave permission for this study to be undertaken in the respective Hospitals. The final decision to participate in the study was taken by the HCWs after reading and understanding the consent form and voluntarily signing it.

## Results

A total of 401 HCWs out of 751 who were invited to participate in the study filled and returned the questionnaire. This led to an overall response rate of 53%. However, if the condition for the required sample is taken into consideration the lower than expected recruitment rate stands at 67%. Non respondents were individuals who were absent during the survey times and days. Most of the study participants were female (67.6%), 45% of them were nurses and 31% non – clinical staff. Only 8% of the studied HCWs had degree level training. Age and type of cadre of HCWs varied systematically with the type of hospital (Table 1).

**Table 1:** **Socio-demographics of healthcare workers (N = 401)**

	**Tumbi (%)**	**Dodoma (%)**	**All (%)**
**Sex:**			
Male	59 (34.5)	71 (30.9)	130 (32.4)
Female	112(65.5)	159 (69.1)	271 (67.6)
**Age:**			
<30 years	39 (22.8)	74 (32.2)*	113 (28.2)
30 – 50 years	106 (62.0)*	111(48.3)	217 (54.1)
>50 years	26 (15.2)	45 (19.6)	71 (17.7)
**Cadre:**			
Clinicians	38 (22.2)*	32 (13.9)	70 (17.5)
Nurses	78 (45.6)	103 (44.8)	181 (45.1)
Other clinical staff	14 (8.2)	10 (4.3)	24 (6.0)
Non-clinical staff	41 (24.0)	85 (37.0)*	126 (31.4)
**Education level**			
Degree	15 (8.8)	17 (7.4)	32 (8.0)
Diploma	72 (42.1)	105 (45.7)	177 (44.1)
Certificate	84 (49.1)	108 (47.0)	192 (47.9)

Chi-Square Test: *p < 0.01.

Over 70% of clinicians and nurses had received education on use of personal protective equipment and on infection prevention and control (Table 2). Generally most of the HCWs were knowledgeable on issues related to recommended measures for prevention of occupational exposure to HIV infections. Methods of prevention of occupational HIV transmission mentioned by HCWs included the use of personal protective gear such as aprons, gloves, goggles and boots, being careful during surgical procedures, handling sharps with care, proper sterilization of re-used instruments, use of safety boxes for proper disposal of needles and sharps, avoiding recapping and decontamination of instruments.

**Table 2:** **Proportion of healthcare workers received education on use of personal protective equipment (PPE), post exposure prophylaxis (PEP) procedure and infection prevention and control (IPC)**

	**Use of PPE**	**IPC education**	**PEP education**
Clinicians	50 (71.4)	55 (78.6)	61 (87.1)
Nurses	135 (74.6)	144 (79.6)	148 (81.8)
Other clinical staff	13 (54.2)	15 (62 5)	14 (58.3)
Non-clinical staff	75 (68.1)	79 (62.7)	75 (59.5)
Total	273 (68.1)	293 (73.1)	298 (74.3)
P - value	0.004	0.016	0.000

Table 3 depicts the proportion of HCWs with comprehensive knowledge on occupational HIV transmission, risk and causes, general issues pertaining to occupational exposure, PEP indication and person to contact in the event of occupational exposure to HIV. Majority of HCWs were well informed on occupational exposure to HIV. Although majority of HCWs (96.3%) knew that they are at risk of occupational HIV transmission, over a quarter of HCWs did not know whom to contact in the event of occupational exposure. Over one third of HCWs did not have comprehensive knowledge on causes of occupational HIV transmission and did not know when PEP is indicated.

**Table 3:** **Proportion of healthcare workers with knowledge on occupational exposure to HIV (N = 401)**

**Knowledge on**	**Tumbi (%)**	**Dodoma (%)**	**All (%)**
Whom to contact in the event of occupational exposure	130 (76.0)	157 (68.3)	287 (71.6)
PEP indication	119 (69.6)	141 (61.3)	260 (64.8)
Risk of occupational HIV transmission possibility	166 (97.1)	220 (95.7)	386 (96.3)
Occupational HIV transmission	151 (83.3)	188 (81.7)	339 (84.5)
Causes of occupational HIV transmission	117 (68.4)	160 (69.6)	277 (69.1)
General knowledge on occupational exposure to HIV	126 (73.7)	164 (71.3)	290 (72.3)
Occupational exposure to HIV (Final knowledge score)	124 (72.5)	151(65.7)	275 (68.6)

Multinomial logistic regression revealed that nurses were more likely to have comprehensive knowledge on occupational exposure to HIV than non-clinical staff (OR = 2.6; 95% CI 1.5 – 4.5); however, this was not the case for Tumbi hospital. HCWs with no training on use of PPE were less likely to have comprehensive knowledge on occupational exposure to HIV (Table 4).

**Table 4:** **Factors influencing occupational exposure to HIV knowledge (Those with comprehensive knowledge in the final knowledge score)**

**Factor**	**Dodoma** ^**a**^	**Tumbi** ^**b**^	**All** ^**c**^
	**OR (95% CI)**	**OR (95% CI)**	**OR (95% CI)**
**Cadre:**			
Clinicians	3.9 (1.1 – 14.0)	0.7 (0.2 – 2.8)	1.9 (0.8 – 4.3)
Nurses	3.8 (1.8 – 7.7)	1.3 (0.5 – 3.4)	2.6 (1.5 – 4.5)
Other clinical staff	1.1 (0.2 – 4.5)	0.7 (0.2 – 3.2)	0.9 (0.3 – 2.4)
Non – clinical staff	1	1	1
**Education on PPE**			
No	0.5 (0.3 – 1.1)	0.9 (0.3 – 2.4)	0.5 (0.3 – 0.9)
Yes	1	1	1
**IEC materials on IPC**			
No	0.8 (0.4 – 1.6)	0.4 (0.2 – 1.1)	0.7 (0.4 – 1.2)
Yes	1	1	
**PEP education**			
No	0.9 (0.4 – 1.9)	0.6 (0.2 – 1.6)	0.8 (0.5 – 1.4)
Yes	1		1
**R** ^**2**^	0.211	0.158	0.152

^a,b^Controlled for age, sex and professional training.

^c^Controlled for age, sex, type of hospital and professional training.

## Discussion

Most of the HCWs studied were nurses, health attendants and medical doctors in that order. This is in line with the pattern of the HCWs employed in the two hospitals. However, with the response rate of 53%, HCWs who participated in the study voluntarily may differ from those who did not respond to the questionnaire. Furthermore, HCWs who participated in the study may not be representative of HCWs employed in these hospitals due to the fact that the minimum sample size required for the study was not reached. Thus the results of this study cannot be generalized to all HCWs in these hospitals.

HCWs studied recognized that they are at risk of occupational HIV transmission in workplaces, similar to reports from other studies [9,10,19,20]. Training on the use of person protective equipment influenced the knowledge of occupational exposure to HIV among studied HCWs. Most clinicians and nurses received training on infection prevention and control and on the use of person protective equipment. This may explain why nurses were more likely to have comprehensive knowledge on occupational exposure to HIV than non-clinical staff.

A substantial proportion of HCWs did not know whom to contact in the event of occupational exposure. Not knowing the contact person may have caused HCWs not to report the event [13]. This increases the risk of HIV transmission as nearly half of the HCWs encounter at least one occupational injury [13].

Over one third of HCWs did not know when PEP is indicated. Similar findings have been reported in a study of HCWs in Dar es Salaam, Tanzania [8]. Studies done in Kathmandu, Malaysia and India indicated that HCWs have either fair or poor knowledge on PEP [21-23]. Optimal post exposure care, including the administration of antiretroviral drugs to prevent HIV infection remains a high priority for protecting health care personnel [24]. It is therefore important that individuals with potential risk of exposure are aware of the procedures to follow and where their first point of contact should be if an incident occurs.

Previous studies conducted in Tumbi and Dodoma hospitals reported approximately 30% of HCWs had poor practice in managing occupational exposure [13] and that measures for preventing occupational exposures to HIV were poorly implemented [15]. This suggests the need for collective efforts to improve awareness of occupational exposure to HIV and its management

This study relied on self-administered questionnaire to assess HCWs knowledge on occupational exposure to HIV. The validity of information in self-reports may be limited by social desirability and recall bias [25]. There is a possibility that in this study socially desired behaviors have been over-estimated and undesired behaviors under-estimated [26,27].

## Conclusion

A substantial proportion of the studied HCWs had little knowledge on occupational exposure to HIV and did not know whom to contact in the event of occupational exposure to HIV. Training on PEP and infection prevention and control including the use of person protective equipment provided to nurses and clinicians should be extended to other clinical and non-clinical hospital staff.

## Abbreviations

AIDS: Acquired immunodeficiency syndrome
HCWs: Health Care Workers
HIV: Human immunodeficiency virus
MoHSW: Ministry of Health and Social Welfare
MRCC: Medical Research Coordinating Committee
NIMR: National Institute for Medical Research
PEP: Post exposure prophylaxis
PPE: Personal protective equipment
TANHER: Tanzania National Health Research Forum
WHO: World Health Organization
